# Dynamic nomogram for predicting axillary lymph node metastasis in breast cancer based on MRI analysis and the platelet-to-lymphocyte ratio (PLR)

**DOI:** 10.1186/s40644-026-01083-z

**Published:** 2026-06-26

**Authors:** Jie Wang, Qinan Geng, Manman Wang, Mengmeng Yang, Yilan Zhang

**Affiliations:** 1 School of Medical Imaging, Jiangsu Vocational College of Medicine, No. 283, Jiefang South Road, Yancheng, Jiangsu 224005 China; 2 Department of Radiology, Yancheng First People’s Hospital, No. 66, Renmin South Road, Yancheng, Jiangsu 224005 China

**Keywords:** Breast cancer, Axillary lymph node metastasis, Platelet-to-lymphocyte ratio (PLR), Nomogram, Magnetic resonance imaging

## Abstract

**Background:**

This study aimed to develop a dynamic nomogram in which clinical indicators are integrated with magnetic resonance imaging (MRI) radiomics to predict axillary lymph node metastasis (ALNM) in patients with breast cancer.

**Methods:**

Our retrospective study included 221 pathologically confirmed patients with breast cancer. Radiomic features were extracted from dynamic contrast-enhanced MRI (DCE-MRI) and fat-suppressed T2-weighted imaging (FS-T2WI) datasets. After feature screening, a support vector machine (SVM) algorithm was employed to establish radiomic models and calculate radiomic scores. Clinical independent predictors were identified through univariate and multivariate logistic regression analyses. A nomogram was established on the basis of the radiomic scores obtained with the optimal SVM model and clinically independent predictors, and it was subsequently transformed into a dynamic nomogram. Model performance was evaluated by the receiver operating characteristic curve and area under the curve (AUC). Shapley additive explanation (SHAP) was applied to interpret the contribution of clinical predictors. Calibration and decision curves were employed to assess nomogram performance.

**Results:**

The platelet-to-lymphocyte ratio (PLR), breast imaging reporting and data system (BI-RADS) classification, and ALN status on MRI were identified as independent predictors of ALNM (all *p* < 0.05). SHAP analysis identified PLR as the top contributor. The clinical model developed with the three predictors achieved AUCs of 0.845 and 0.706 in the training and validation cohorts, respectively. Among the SVM models, the DCE-MRI and FS-T2WI fusion sequence model outperformed the single-sequence models, with AUCs of 0.868 and 0.875, respectively, in the training and validation cohorts. When clinical predictors were incorporated into the fusion sequence model, the nomogram achieved AUC values of 0.930 and 0.928 in the training and validation cohorts. Decision curve analysis demonstrated that the nomogram has significant clinical value.

**Conclusions:**

A nomogram model integrating MRI-derived radiomic scores, BI-RADS classification, ALN status, and PLR demonstrated good performance in predicting ALNM in patients with breast cancer.

**Supplementary Information:**

The online version contains supplementary material available at https://doi.org/10.1186/s40644-026-01083-z.

## Background

According to the Cancer Statistics 2025 report, breast cancer accounts for 32% of the total number of new diagnoses among women. The incidence of breast cancer is continuously increasing, with an average annual increase of 1% [1]. Metastasis to axillary lymph nodes (ALNs) is crucial to the clinical staging, prognosis assessment and personalized treatment of breast cancer [2]. In traditional breast cancer treatment paradigms, mastectomy combined with axillary lymph node dissection (ALND) is widely regarded as the standard therapeutic approach. However, patients who undergo ALND may experience a range of postoperative complications, which can adversely affect their postoperative quality of life [3]. Currently, sentinel lymph node biopsy (SLNB) has gradually replaced ALND as the standard procedure for ALN assessment in patients with breast cancer because of its minimally invasive nature and reduced complication risk. However, SLNB carries the potential for high false-negative rates and may cause minor damage to blood vessels and nerves [4]. Therefore, for patients without ALN metastasis (ALNM), there is an urgent need for a safe and noninvasive assessment method to evaluate the ALN status, thereby avoiding invasive procedures and ultimately improving patient prognosis and quality of life.

Magnetic resonance imaging (MRI) plays a vital role in determining the accuracy of breast cancer diagnosis, facilitating the effective localization of tumor cells and assessment of their infiltration and spread [5]. MRI has become an effective auxiliary method for breast cancer diagnosis. Dynamic contrast-enhanced MRI (DCE-MRI) can provide information on tumor vascular distribution and blood perfusion through the observation of the dynamic enhancement characteristics of tumors after contrast agent injection [6, 7]. Recent studies have demonstrated that radiomics can significantly enhance medical imaging diagnostic performance by extracting deep image features, thereby overcoming the limitations of traditional morphological diagnosis and revealing the deep heterogeneity of tumors [8, 9]. Radiomic analysis of breast tumors is widely applied in evaluating the pathological grade [10], ALNM [11], molecular classification [12], and response to neoadjuvant chemotherapy [13]. However, no prior studies on ALNM radiomics have incorporated serum inflammatory factors.

Inflammation constitutes an important component of the tumor microenvironment. The inflammatory response affects the proliferation, invasion and metastasis of tumor cells, and systemic inflammatory markers are closely related to the risk and mortality of cancer [14, 15]. In routine blood tests, the neutrophil-to-lymphocyte ratio (NLR), platelet-to-lymphocyte ratio (PLR), and lymphocyte-to-monocyte ratio (LMR) are the most common indicators of systemic inflammation. Previous studies have confirmed that the PLR can serve as a predictor of lymph node metastasis in patients with breast cancer [16, 17].

Herein, we hypothesized that the preoperative PLR may vary depending on the ALN status in patients with breast cancer. In this study, we developed a nomogram in which PLR and MRI features using radiomics are combined, aiming to provide a noninvasive and preoperative ALNM assessment solution for breast cancer patients.

## Methods

### Patients

This study aimed to retrospectively analyse patients with breast cancer confirmed by surgical pathology at Yancheng First People’s Hospital from January 2021 to December 2024. The inclusion criteria for patients were as follows: (1) pathologically confirmed breast cancer with an ALN stage determined through SLNB or ALND; (2) no prior treatment; (3) preoperative MRI performed within two weeks, with complete sequences and clear images; and (4) routine blood test conducted one week before surgery. The exclusion criteria were as follows: (1) previous procedures such as biopsy or neoadjuvant therapy; (2) small tumor size or poor MR image quality affecting region of interest (ROI) delineation; and (3) incomplete clinical and pathological data. This study was approved by the hospital ethics committee (2022-k135).

A total of 221 patients were ultimately included in the study. They were divided into a training cohort and a validation cohort at a ratio of 7:3. The breast cancer patients with ALNM were classified into the positive group (ALN+), while those without ALNM were classified into the negative group (ALN-).

### MRI procedure and analysis

All patients underwent examination via the use of 3.0 T Siemens MRI scanners with 8-channel breast coils.

MRI protocols included T1-weighted imaging (T1WI), fat-suppressed T2-weighted imaging (FS-T2WI), diffusion-weighted imaging (DWI), and DCE-MRI. After mask application, contrast agent Gd-DTPA (Bayer, Germany) was injected via the antecubital vein at a dose of 0.1 mmol/kg and a flow rate of 2.5 ml/s, followed by a 20 ml saline flush administered at the same rate. Images were collected in a total of 6 phases, each lasting 60 s. Further parameters are detailed in supplementary Table S1.

In this study, all imaging analyses were independently performed by two physicians with more than eight years of diagnostic experience in breast MRI who were blinded to the pathological results. The inclusion criteria included tumor size (maximum diameter), tumor morphology (regular or irregular), enhancement pattern (mass-like or non-mass-like), time-intensity curve (TIC) type (inflow type I, plateau type II, or outflow type III), breast imaging reporting and data system (BI-RADS) classification based on MRI (< 4, 4a, 4b, 4c, or 5), and ALN status on MRI (MRI-ALN status, [negative or positive]). A positive diagnosis required any of the following: (1) short-axis lymph node diameter > 1 cm; (2) lateral cortex thickening over 4 mm; and (3) absence of a fat hilum [3]. Disputes were resolved through discussion.

### Haematological and pathological data

After MRI and routine blood tests were performed, the ALN status was determined through the use of SLNB or ALND, with histological examination conducted by a pathologist with 10 years of experience. ALNM was defined as the presence of micrometastasis (> 0.2 mm, ≤ 2.0 mm) or macrometastasis (> 2.0 mm) [18].

Haematologic data included lymphocyte, neutrophil, monocyte, and platelet counts. The following composite indicators were calculated: the LMR, NLR, PLR, and systemic immune inflammation index (SII) = platelet × neutrophil/lymphocyte ratio. Baseline data included age, tumor location, and tumor quadrant.

The pathological data included estrogen receptor (ER) status, progesterone receptor (PR) status, human epidermal growth factor receptor 2 (HER2) status, histological grade, and Ki-67%. Immunohistochemistry (IHC) was used to determine the molecular receptor status and Ki-67 expression. The positive criterion for ER and PR expression was defined as 1%. HER2 was classified as positive (IHC 3 + or IHC 2 + confirmed by a fluorescence in situ hybridization (FISH) amplification test) or negative (IHC 0, 1+, or 2 + with a negative FISH result) [19].

### Image preprocessing and segmentation

**Fig. 1:**
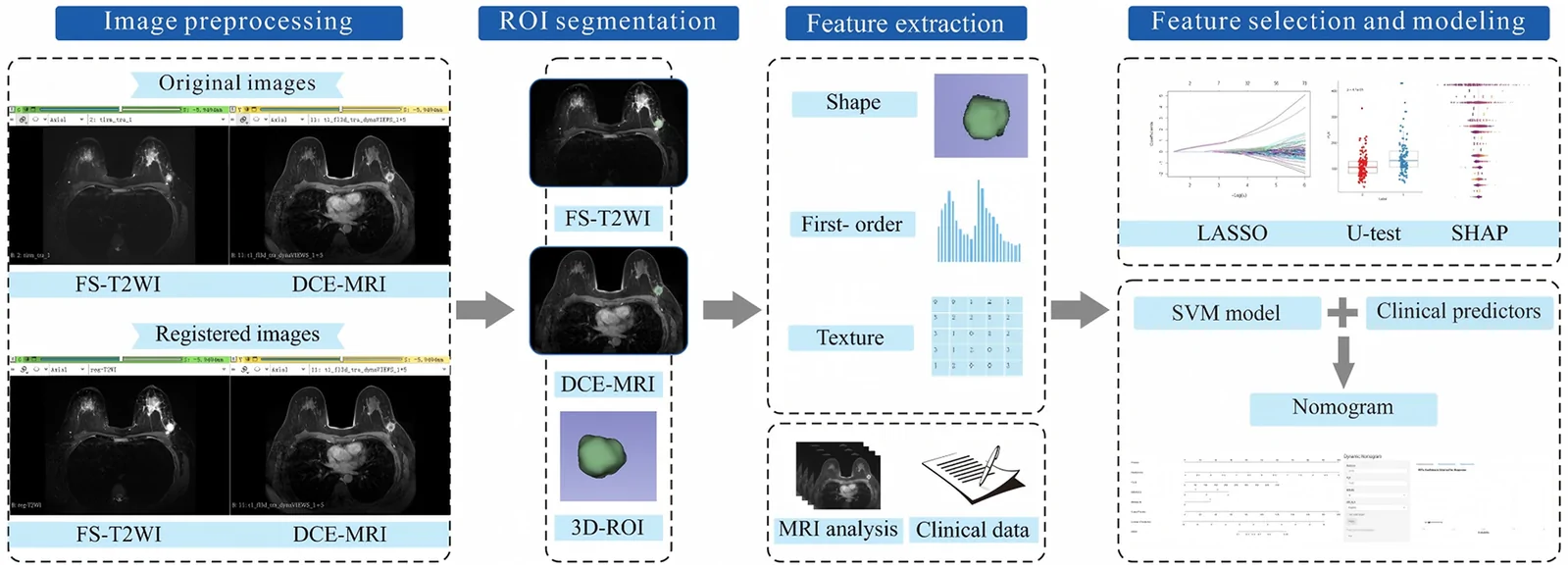
Schematic workflow of the study

All image ROI delineation and feature extraction were performed using the open source software 3DSlicer 5.0.3, as illustrated in Fig. 1. Before image segmentation, all images underwent preprocessing via the N4MRI bias correction module to eliminate artefacts and ensure uniformity. The DCE-MRI and FS-T2WI were then registered by Elastix. ROIs were initially identified by a researcher through manual recognition on the second enhancement phase of DCE-MRI [3]. A region-growing segmentation algorithm was then applied to automatically connect homogenous or similar voxels, achieving semiautomatic ROI delineation. The ROIs were subsequently automatically mapped to the FS-T2WI scan and manually adjusted for optimization. After a senior radiologist confirmation, the ROI fully encompassed the tumor, including areas of enhancement and necrosis, while avoiding adjacent blood vessels and normal tissues. Tumor boundaries were progressively defined to ultimately generate a full-tumor 3D ROI. In cases with multiple lesions, the largest single lesion was selected.

### Radiomic features extraction and selection

The Radiomics module was used to sequentially extract features from each sequence, with 944 features per sequence, including shape features, first-order statistical features, texture features, and features based on the wavelet or Laplacian of Gaussian, from one ROI. One month later, 30 patients were randomly selected for ROI redelineation and feature extraction by the same radiologist and another senior radiologist. The intra- and inter- observer correlation coefficients (ICC) were calculated to assess feature reproducibility, with values > 0.75 indicating good consistency.

All features were subjected to z score normalization to quantify and weight parameters across different units. With respect to DCE-MRI and FS-T2WI radiomic features screening, variables with a high linear correlation (cut-off = 0.9) were removed to prevent multicollinearity by Pearson analysis. The least absolute shrinkage and selection operator (LASSO) algorithm was subsequently employed to eliminate zero-coefficient features. The optimal regularization strength λ value was determined using the 10-fold cross-validation, with the minimization of mean squared error (MSE) as the selection criterion. The lambda.min was adopted as the final hyperparameter. By selecting linear combinations of these features and weighting their coefficients, the radiomic score (Radscore) of each patient was calculated.

### Model and nomogram construction

All clinical indicators were compared between the ALN- and ALN+ groups. Univariate and multivariate logistic regression analyses were performed to identify significant ALNM-related factors, with only variables for which *p* was < 0.05 in the univariate analysis being included in the multivariate analysis. To analyze and quantify the contribution of clinical indicators to the logistic regression, we employed the Shapley additive explanation (SHAP). Clinical prediction model was constructed using these significant risk factors by logistic regression algorithm.

We developed the following three support vector machine (SVM) algorithm models: one for FS-T2WI, one for DCE-MRI, and another for fusion sequence. The kernel function for SVM was set to “linear” and the penalty parameter C was 1. Finally, a multimodality model integrating Radscores derived from the optimal SVM model and clinically significant factors was developed to construct a nomogram. An online version of the nomogram tool was also developed. Clinicians can input relevant parameters to calculate the probability of ALNM in real time.

### Statistical analysis

SPSS (version 26.0), R (version 4.5.1, “tidyverse”, “ggplot2”, “caret”, “glmnet”, “e1071”, “pROC”, “rmda”, “rms”, “shapviz”, “DynNom”, “rsconnect” packages) and Medcalc (version 22.0) were used for statistical analysis. Categorical data are described as frequencies and percentages. Continuous variables with a normal distribution are expressed as the mean±standard deviation, while other variables are presented as the median and interquartile range. To analyse group differences, Student’s t test or the Mann–Whitney U test was used for continuous variables, and the chi-square test or Fisher’s exact test was used for categorical variables. *p* < 0.05 was considered to indicate a statistically significant difference.

Receiver operating characteristic (ROC) curves were employed to assess the diagnostic performance of each model visually, which yielded key metrics, including the area under the curve (AUC), accuracy (ACC), sensitivity (SEN), specificity (SPE), positive predictive value (PPV), and negative predictive value (NPV). The Delong test was employed to assess diagnostic performance differences between the models. Calibration curves and the Hosmer–Lemeshow test were used to validate the performance of the nomogram. Decision curve analysis (DCA) was conducted to quantify the net benefit of the different models and evaluate their clinical value.

## Results

### Baseline data

Among the 221 patients, 124 patients were classified into the ALN- group, and 97 patients were classified into the ALN+ group. The patients were divided into a training cohort (*n* = 155) and a validation cohort (*n* = 66) at a 7:3 ratio. All features did not significantly differ between the training and validation cohorts (all *p* > 0.05) (Supplementary Table S2). In terms of clinical characteristics, no statistically significant differences were observed between the ALN- and ALN+ groups in terms of age, tumor location, ER, PR, or HER2 expression, histological grade, or neutrophil, lymphocyte, and monocyte count (*p >* 0.05). However, the tumor quadrant, platelet count, LMR, NLR, PLR, SII, and Ki-67% significantly differed between the two groups (*p* < 0.05). In terms of MRI features, notable differences in tumor size, BI-RADS classification, and MRI-ALN status were detected between the ALN- and ALN+ groups (*p* < 0.05), whereas no differences were detected in tumor morphology, enhancement pattern, or TIC (*p >* 0.05), as detailed in Table 1.

**Table 1:** Comparison of clinical and MRI features between ALN- and ALN+ groups

Characteristics	ALN- group(*n* = 124)	ALN+ group(*n* = 97)	*P*
Age [year, mean ± SD]	48.61 ± 9.68	50.09 ± 11.78	0.346
Tumor location			0.207
Left	68 (54.8%)	44 (45.4%)	
Right	56 (45.2%)	53 (54.6%)	
Tumor quadrant			0.001
Outer upper	39 (31.5%)	28 (28.9%)	
Outer lower	33 (26.6%)	25 (25.8%)	
Upper inner	28 (22.6%)	14 (14.4%)	
Lower inner	19 (15.3%)	9 (9.3%)	
Others	5 (4.0%)	21 (21.6%)	
Lymphocyte [×10^9^/L, *M*(*P*_25_, *P*_75_)]	1.78 (1.42, 2.07)	1.73 (1.35, 2.04)	0.258
Neutrophil [×10^9^/L, *M*(*P*_25_, *P*_75_)]	3.14 (2.52, 3.81)	3.48 (2.88, 4.17)	0.081
Monocyte [×10^9^/L, *M*(*P*_25_, *P*_75_)]	0.41 (0.33, 0.50)	0.43 (0.35, 0.49)	0.488
Platelet [×10^9^/L, mean ± SD]	199.99 ± 64.54	219.44 ± 64.64	0.020
LMR [*M*(*P*_25_, *P*_75_)]	4.43 (3.69, 5.53)	4.22 (3.21, 5.10)	0.029
NLR [*M*(*P*_25_, *P*_75_)]	1.60 (1.35, 2.21)	1.98 (1.49, 2.60)	0.016
PLR [*M*(*P*_25_, *P*_75_)]	104.89 (81.99, 128.54)	129.84 (105.83, 166.96)	< 0.001
SII [*M*(*P*_25_, *P*_75_)]	330.25 (238.19, 417.69)	419.96 (324.24,569.55)	< 0.001
ER			0.199
Negative	47 (37.9%)	46 (47.4%)	
Positive	77 (62.1%)	51 (52.6%)	
PR			0.087
Negative	46 (37.1%)	48 (49.5%)	
Positive	78 (62.9%)	49 (50.5%)	
HER2			0.478
Negative	86 (69.4%)	62 (63.9%)	
Positive	38 (30.6%)	35 (36.1%)	
Histological grade			0.471
Ⅰ	36 (29.0%)	22 (22.7%)	
Ⅱ	58 (46.8%)	46 (47.4%)	
Ⅲ	30 (24.2%)	29 (29.9%)	
Ki-67% [*M*(*P*_25_, *P*_75_)]	30.00 (10.00, 42.50)	40.00 (30.00, 50.00)	< 0.001
Tumor size [mm, *M*(*P*_25_, *P*_75_)])	23.18 (16.57, 31.69)	30.12 (21.07, 42.14)	< 0.001
Tumor morphology			0.053
Regular	62 (50%)	35 (36.1%)	
Irregular	62 (50%)	62 (63.9%)	
Enhanced pattern			0.651
Mass-like	84 (67.7%)	62 (63.9%)	
Non-mass-like	40 (32.3%)	35 (36.1%)	
TIC			0.219
Ⅰ	8 (6.5%)	3 (3.1%)	
Ⅱ	42 (33.9%)	26 (26.8%)	
Ⅲ	74 (59.7%)	68 (70.1%)	
BI-RADS			< 0.001
<4	6 (4.8%)	4 (4.1%)	
4a	34 (27.4%)	7 (7.2%)	
4b	31 (25.0%)	16 (16.5%)	
4c	33 (26.6%)	32 (33.0%)	
5	20 (16.1%)	38 (39.2%)	
MRI-ALN			< 0.001
Negative	107 (86.3%)	51 (52.6%)	
Positive	17 (13.7%)	46 (47.4%)	

ALN, axillary lymph node, LMR Lymphocyte monocyte ratio, NLR Neutrophil lymphocyte ratio, PLR Platelet lymphocyte ratio, SII Systemic immune inflammation index, ER oestrogen receptor, PR progesterone receptor, HER2 human epidermal growth factor receptor 2, TIC Time-signal intensity curve, BI-RADS Breast imaging reporting and data system, MRI-ALN ALN status on MRI

### Radiomic features selection

In this study, a total of 1888 radiomics features were extracted from each ROI by FS-T2WI (*n* = 944) and DCE-MRI (*n* = 944). The fusion sequence combined the features of FS-T2WI and DCE-MRI. In the training cohort, 255, 249, and 484 robust features were retained from the FS-T2WI, DCE-MRI, and fusion sequence after Pearson’s correlation analysis. The LASSO algorithm was then applied to filter the features, ultimately retaining 5 FS-T2WI, 9 DCE-MRI, and 5 fusion sequence features, with all the coefficients remaining nonzero. The selected features and feature coefficients were detailed in Supplementary Fig. 1.

### Model construction and evaluation

On the basis of the selected features, we constructed three independent radiomic models (FS-T2WI, DCE-MRI, and fusion sequence) via the SVM algorithm. These models achieved AUCs of 0.824, 0.853, and 0.868 in the training cohort and 0.778, 0.858, and 0.875 in the validation cohort, respectively. The diagnostic performance slightly increased when the FS-T2WI and DCE-MRI sequences were combined.

**Fig. 2:**
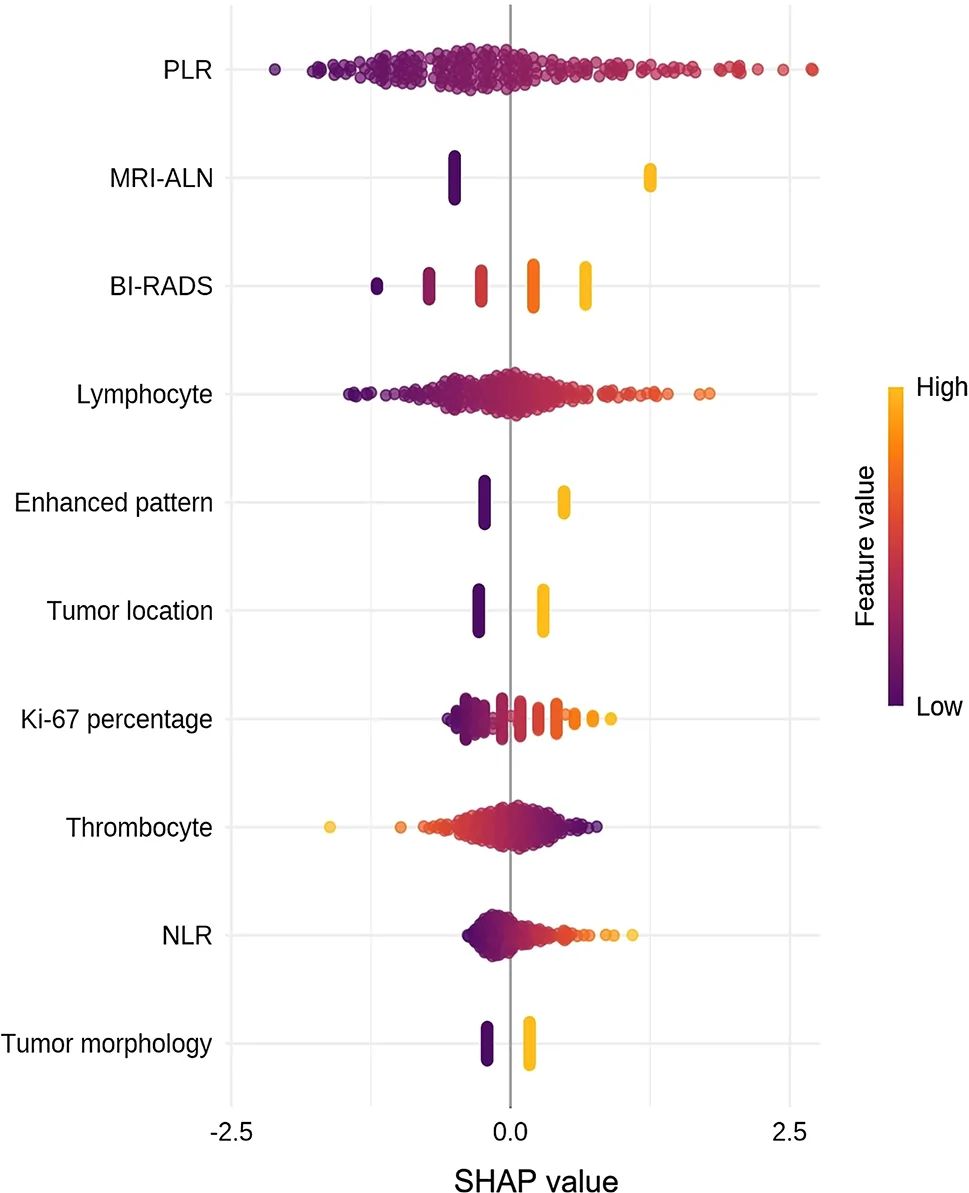
SHAP plot of the clinical model shows the impact of each feature on the model output

The results of univariate logistic regression analysis revealed that the platelet count, NLR, PLR, SII, Ki-67%, tumor size, BI-RADS classification, and MRI-ALN status were all potential predictors of ALNM (all *p* < 0.05). Multivariate analysis further confirmed that the PLR (odds ratio [OR] = 1.010, 95% CI: 1.001, 1.019; *p* = 0.039), BI-RADS classification (OR = 1.504, 95% CI: 1.133, 2.020; *p* = 0.005), and MRI-ALN status (OR = 4.608, 95% CI: 2.267, 9.722; *p* < 0.001) were independent predictors of ALNM (Table 2). The feature importance analysis based on SHAP showed that PLR, MRI-ALN status and BI-RADS classification were the most influential features in the logistic regression algorithm (Fig. 2). Notably, PLR had the highest mean SHAP value (0.925). Therefore, these three indicators were used to construct a clinical model and incorporated into the nomogram model.

**Table 2:** Univariate and multivariate logistic regression analysis of characteristics associated with breast cancer axillary lymph node metastasis

Characteristics	Univariate analysis		Multivariate analysis		
	Odds ratio (95%% CI)	*P* value	β coefficient	Odds ratio (95%% CI)	*P* value
Age	1.013 (0.988–1.039)	0.306			
Tumor location	1.462 (0.858–2.502)	0.163			
Tumor quadrant	1.215 (0.996–1.485)	0.055			
Lymphocyte	0.734 (0.448–1.173)	0.205			
Neutrophil	1.172 (0.966–1.441)	0.115			
Monocyte	1.392 (0.194–10.038)	0.741			
Platelet	1.005 (1.001–1.009)	0.029*	-0.001	0.999 (0.989–1.007)	0.911
LMR	0.929 (0.795–1.056)	0.305			
NLR	1.436 (1.064–1.968)	0.020*	0.092	1.096 (0.459–1.954)	0.793
PLR	1.011(1.006–1.017)	< 0.001*	0.009	1.010 (1.001–1.019)	0.039*
SII	1.002 (1.001–1.003)	< 0.001*	0.001	1.000 (0.997–1.004)	0.783
ER	0.676 (0.393–1.159)	0.156			
PR	0.602 (0.349–1.031)	0.065			
HER2	1.277 (0.726–2.247)	0.394			
Histological grade	1.257 (0.871–1.821)	0.223			
Ki-67%	1.020 (1.001–1.034)	0.003*	0.009	1.009 (0.993–1.025)	0.253
Tumor size	1.034 (1.017–1.054)	< 0.001*	0.016	1.016 (0.996–1.038)	0.125
Tumor morphology	1.771 (1.032–3.068)	0.052			
Enhanced pattern	1.185 (0.676–2.076)	0.551			
TIC	1.518 (0.954–2.467)	0.084			
BI-RADS	1.770 (1.385–2.298)	< 0.001*	0.409	1.504 (1.133–2.020)	0.005*
MRI-ALN	5.677 (3.017–11.103)	< 0.001*	1.528	4.608 (2.267–9.722)	< 0.001*

*highlights the *p* values that are smaller than 0.05

### Nomogram evaluation

**Fig. 3:**
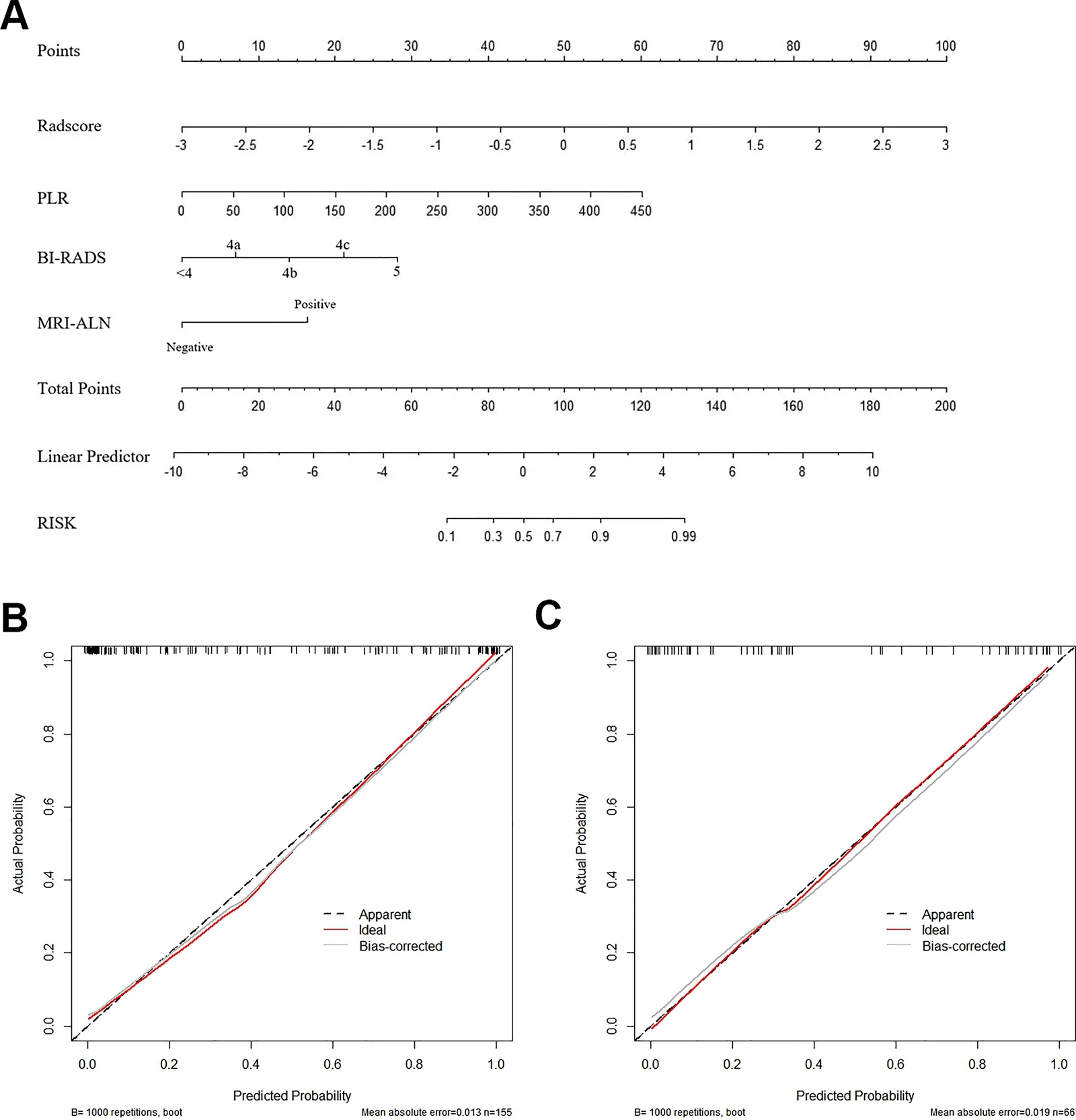
A nomogram for predicting ALNM (**A**). The calibration curves of nomogram in the training (**B**) and validation (**C**) cohorts. The closer the curves are to the diagonal dashed line, the higher the predictive performance of the model

**Fig. 4:**
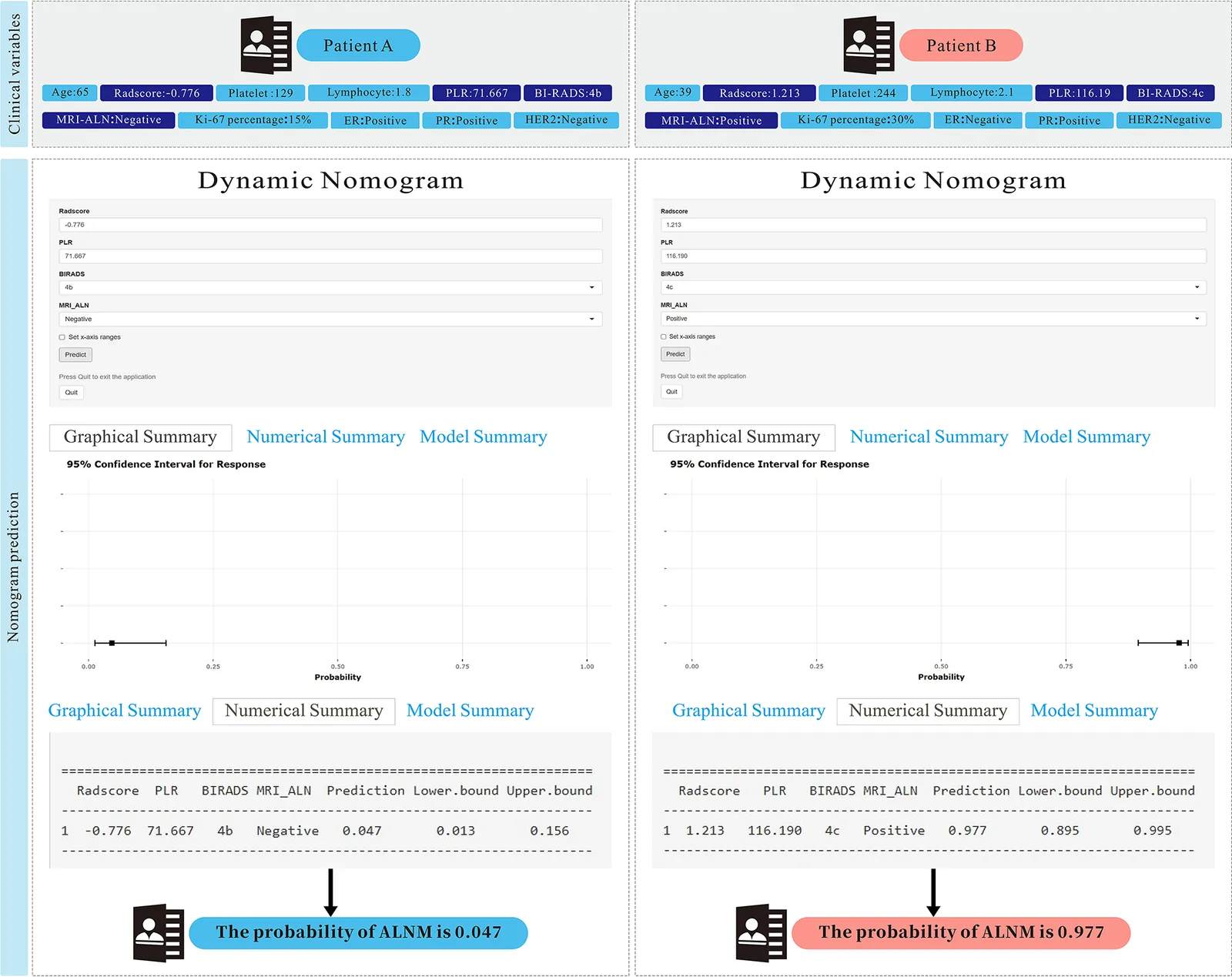
This schematic illustrates the application of dynamic nomogram in predicting ALNM in two breast cancer patients. When the Radscore, PLR, and MRI-ALN of patients A and B were incorporated into the nomogram model, the probability of ALNM in patient A was 4.7%, while that in patient B was 97.7%. Pathological confirmation revealed that patient A had no ALNM, whereas patient B presented with ALNM

**Fig. 5:**
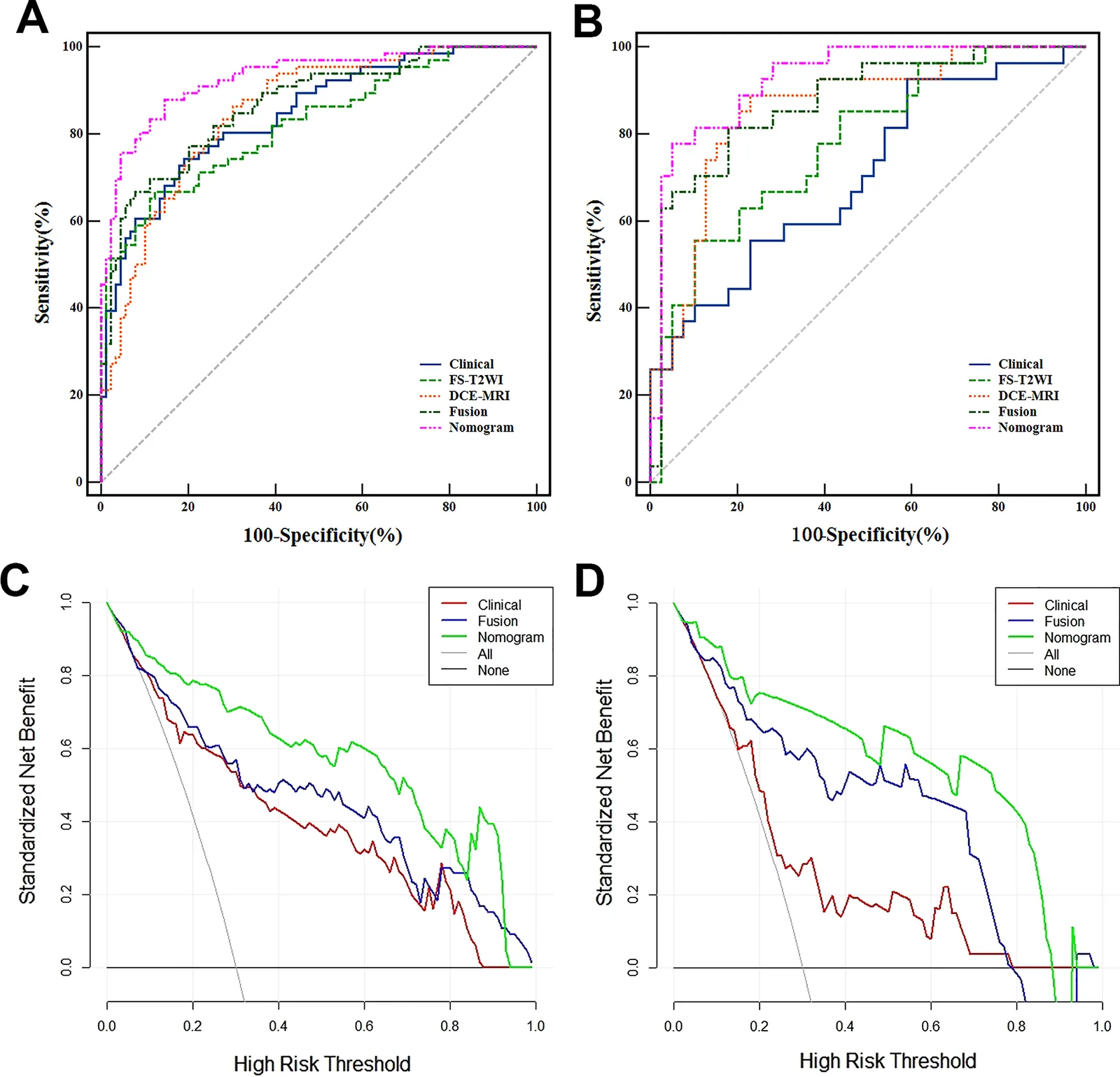
The ROC curves of different models predicting ALNM in training (**A**) and validation (**B**) cohorts. Decision curve analysis evaluated the clinical utility of the models in the training (**C**) and validation (**D**) cohorts

A nomogram (Fig. 3A) was developed using the PLR, BI-RADS classification, MRI-ALN status, and Radscores from the fusion sequence radiomic model, which demonstrated significantly improved diagnostic performance. The AUCs for the training and validation cohorts were 0.930 and 0.928, respectively (Table 3). Delong’s test revealed statistically significant differences in the AUC value between the nomogram and the fusion sequence model, as well as between the nomogram and the clinical model (z = -3.178, *p =* 0.001; z = -3.194, *p =* 0.001). The calibration curve demonstrated good consistency between the actual and theoretical predictions (Fig. 3B-C). The Hosmer–Lemeshow test indicated no statistically significant difference between the predicted outcomes and actual incidence probabilities in both the training and validation cohorts, confirming a strong model fit (*p* = 0.909 and 0.759, respectively). Additionally, a dynamic nomogram was successfully created (https://alnnomogram.shinyapps.io/dynnomapp/). Patients with high Radscores, PLRs, and BI-RADS classifications, as well as MRI-ALN positivity were more likely to develop ALNM (Fig. 4). ROC and decision curves analysis revealed that the nomogram outperformed both the clinical model and the fusion sequence model, highlighting its superior clinical application value (Fig. 5A-D).

**Table 3:** The performance of different evaluation approaches for predicting axillary lymph node metastasis in training and validation cohorts

Cohort	Approach	AUC(95%CI)	ACC(%)	SEN(%)	SPE(%)	PPV(%)	NPV (%)
Training	Clinical	0.845 (0.784, 0.907)	78.06	74.15	77.28	74.24	80.89
	FS-T2WI	0.824 (0.757, 0.891)	78.71	73.03	72.73	80.00	78.00
	DCE-MRI	0.853 (0.795, 0.912)	76.77	82.02	71.22	67.85	87.32
	Fusion sequence	0.868 (0.811, 0.925)	81.29	88.76	69.70	86.27	78.85
	Nomogram	0.930 (0.890, 0.971)	86.45	91.01	80.31	81.69	90.48
Validation	Clinical	0.706 (0.576, 0.835)	62.12	76.92	55.66	52.08	88.89
	FS-T2WI	0.778 (0.664, 0.891)	75.76	76.92	62.97	78.94	74.47
	DCE-MRI	0.858 (0.765, 0.952)	81.82	84.61	77.78	72.72	90.91
	Fusion sequence	0.875 (0.786, 0.963)	81.82	82.05	81.48	75.86	86.48
	Nomogram	0.928 (0.866, 0.990)	87.88	89.74	81.48	91.30	86.04

AUC area under curve, CI confdence interval, ACC accuracy, SEN sensitivity, SPE specifcity, PPV positive predictive value, NPV negative predictive value

## Discussion

Currently, there is no noninvasive and convenient method for accurately predicting ALNM in patients with breast cancer. In this study, we developed a nomogram integrating radiomic features and clinical indicators to predict ALNM. The model achieved excellent AUCs of 0.930 in the training cohort and 0.928 in the validation cohort, outperforming the SVM and clinical models.

Furthermore, we developed a dynamic nomogram to visualize statistical models for interactive applications, enabling clinicians to assess the risk of ALNM in breast cancer patients.

The ALN status serves as a critical clinical guide, as ALNM typically indicates a poor prognosis and high recurrence risk. Studies have shown that MRI-based radiomics can effectively predict the ALN status, but most studies have focused on DCE-MRI-based radiomic analysis [4, 20, 21]. Compared with the DCE-MRI-based model developed by MAO et al. (with AUCs of 0.78 and 0.79 in the training and validation cohorts, respectively), the radiomics model established in this study incorporated both DCE-MRI and FS-T2WI and demonstrated superior diagnostic performance [11]. This advantage likely stems from the significant value of FS-T2WI in evaluating breast masses, as it clearly reveals oedema, haemorrhage, and cystic necrosis [22]. In addition, DWI is a widely used and highly effective clinical technique for evaluating breast lesions [23]. Dong et al. pioneered the use of radiomics combining FS-T2WI and DWI to predict the ALN status in patients. Their study revealed that the AUC of the combined model in the validation group was 0.805, which was lower than the 0.875 reported in this research [24]. On DWI, most lesions appear blurred with low resolution, which poses challenges for precise segmentation [25]. Therefore, DWI was not included in this study. DCE-MRI can be used to evaluate angiogenesis and reflect blood flow within tumors [26], whereas FS-T2WI is better for detecting abnormal signals such as tumor margins and oedema. Multimodal radiomic models based on fusion sequence can improve diagnostic accuracy and guide the development of clinical treatment plans.

The development of tumors is influenced by chronic inflammation and immune responses in the tumor microenvironment. The NLR, PLR, and LMR reflect the relative balance between tumor-associated inflammation and antitumor immune responses [27, 28]. In our research, the PLR served as an independent predictor of ALNM, with the ALN+ group exhibiting a significantly higher PLR than the ALN- group did. Previous studies have shown that an elevated PLR is linked to clinical outcomes in patients with breast cancer [29]. For patients receiving neoadjuvant chemotherapy (NACT), a higher PLR correlated with lower rates of pathological complete response, as well as poorer overall survival (OS) and disease-free survival (DFS) [30], indicating that the PLR is a valuable prognostic marker for breast cancer. Furthermore, a meta-analysis of 20 studies revealed that breast cancer patients with a higher PLR were more likely to develop ALNM (OR = 1.82, 95% CI: 1.32, 2.52, *p* < 0.001) and distant metastases than those with a lower PLR [31]. Wang et al. also reported that an elevated PLR was associated with an increased risk of ALNM [17]. These study findings are highly consistent with the findings of this research. During inflammation, the imbalance between platelets and lymphocytes elevates the PLR, with platelets aggregating around tumor cells to resist immune attacks [29]. Platelets can enhance the adhesion between tumor cells and endothelial cells, thereby increasing the permeability of endothelial cells and promoting the extravasation of tumor cells. Remarkably, these platelets secrete immunosuppressive factors such as vascular endothelial growth factor (VEGF), which induces angiogenesis and promotes tumor growth and metastasis [32]. Lymphocytes can effectively suppress the invasion, migration, and proliferation of tumor cells through the synergistic actions of CD8 + T cells-mediated targeted killing, B cells-dependent antigen presentation and antibody secretion, as well as NK cell-mediated innate immune surveillance. A decrease in lymphocyte count may lead to insufficient immune response, thus adversely affecting the prognosis of patients with malignant tumors [33]. In recent years, the PLR has been shown to be closely associated with clinical prognosis and treatment efficacy in various cancers [34–36].

In our study, the MRI-ALN status and BI-RADS classification were also identified as independent predictors of ALNM. Chen et al. [4] demonstrated through multivariate logistic regression analysis that the ALN status on MRI reports significantly predicts ALNM in patients with breast cancer. After incorporating this information into the nomogram, the AUC increased from 0.86 to 0.90. This finding was also corroborated by another study [3]. By analysing multiple clinical and histopathological features, Zhu et al. reported that only the BI-RADS classification and tumor size significantly differed between the ALN- and ALN+ groups (*p* < 0.01). Additionally, they constructed five machine learning models, with SVM achieving the greatest diagnostic performance (AUC = 0.86) [37]. Notably, unlike the findings of previous studies [37–39], our findings revealed that the tumor size significantly differed in the univariate analysis but not in the multivariate analysis. This discrepancy may be attributed to differences in sample size and demographic characteristics of the study population. This study comprehensively revealed tumor heterogeneity by combining complementary DCE-MRI and FS-T2WI radiomic data. When clinical risk factors were incorporated, the AUC of the prediction model for breast cancer ALNM improved from 0.875 to 0.928 in the validation cohort. On this basis, a dynamic nomogram was developed to integrate multiple datasets for ALN status prediction. Numerous breast cancer ALNM nomograms utilizing MRI radiomics have been constructed, yet they are frequently limited to single sequences or neglect haematological inflammatory biomarkers [3, 11, 40].

This study exhibits several limitations. Firstly, our ROI delineation covered the intratumoral area, and future research should focus on supplementary studies of the peritumoral region. Secondly, the retrospective single centre design may introduce selection bias, and the stability and reproducibility of the model lack external validation. Although our research demonstrated considerable predictive efficacy, it is still necessary to use data from multiple centers with larger sample size to verify the model. Thirdly, the analysis utilized only the second enhancement phase of DCE-MRI, resulting in missing tumor biological information. More phases can be included in future studies.

## Conclusions

In conclusion, we developed a predictive ALNM dynamic nomogram model by integrating the Radscore, BI-RADS classification and ALN status derived from MRI analysis, along with the PLR. This model may provide valuable evidence for clinical decision-making in breast cancer patients.

## Supplementary Information

Below is the link to the electronic supplementary material.

Supplementary Material 1

## Data Availability

No datasets were generated or analysed during the current study.

## Abbreviations

ACC: Accuracy
ALN: Axillary lymph node
ALND: Axillary lymph node dissection
ALNM: Axillary lymph node metastasis
AUC: The area under the curve
BI-RADS: The breast imaging reporting and data system
DCA: Decision curve analysis
DCE-MRI: Dynamic contrast-enhanced MRI
DWI: Diffusion-weighted imaging
ER: Estrogen receptor
FS-T2WI: Fat-suppressed T2-weighted imaging
HER2: Human epidermal growth factor receptor 2
ICC: The intraclass correlation coefficient
LASSO: The least absolute shrinkage and selection operator
LMR: Lymphocyte-to-monocyte ratio
MRI: Magnetic resonance imaging
MRI-ALN: Axillary lymph node status on magnetic resonance imaging
NLR: Neutrophil-to-lymphocyte ratio
NPV: Negative predictive value
PLR: Platelet to lymphocyte ratio
PPV: Positive predictive value
PR: Progesterone receptor
ROC: Receiver operating characteristic
SEN: Sensitivity
SHAP: Shapley additive explanation
SLNB: Sentinel lymph node biopsy
SPE: Specificity
SVM: Support vector machine
T1WI: T1-weighted imaging
TIC: Time-intensity curve
