# Supplementary material for: Dynamic nomogram for predicting axillary lymph node metastasis in breast cancer based on MRI analysis and the platelet-to-lymphocyte ratio (PLR)
Source: Cancer Imaging. 2026 Jun 26;26:135. doi: 10.1186/s40644-026-01083-z (PMC13595517; doi:10.1186/s40644-026-01083-z)
Supplement: Supplementary file 1 [file 40644_2026_1083_MOESM1_ESM.pdf]

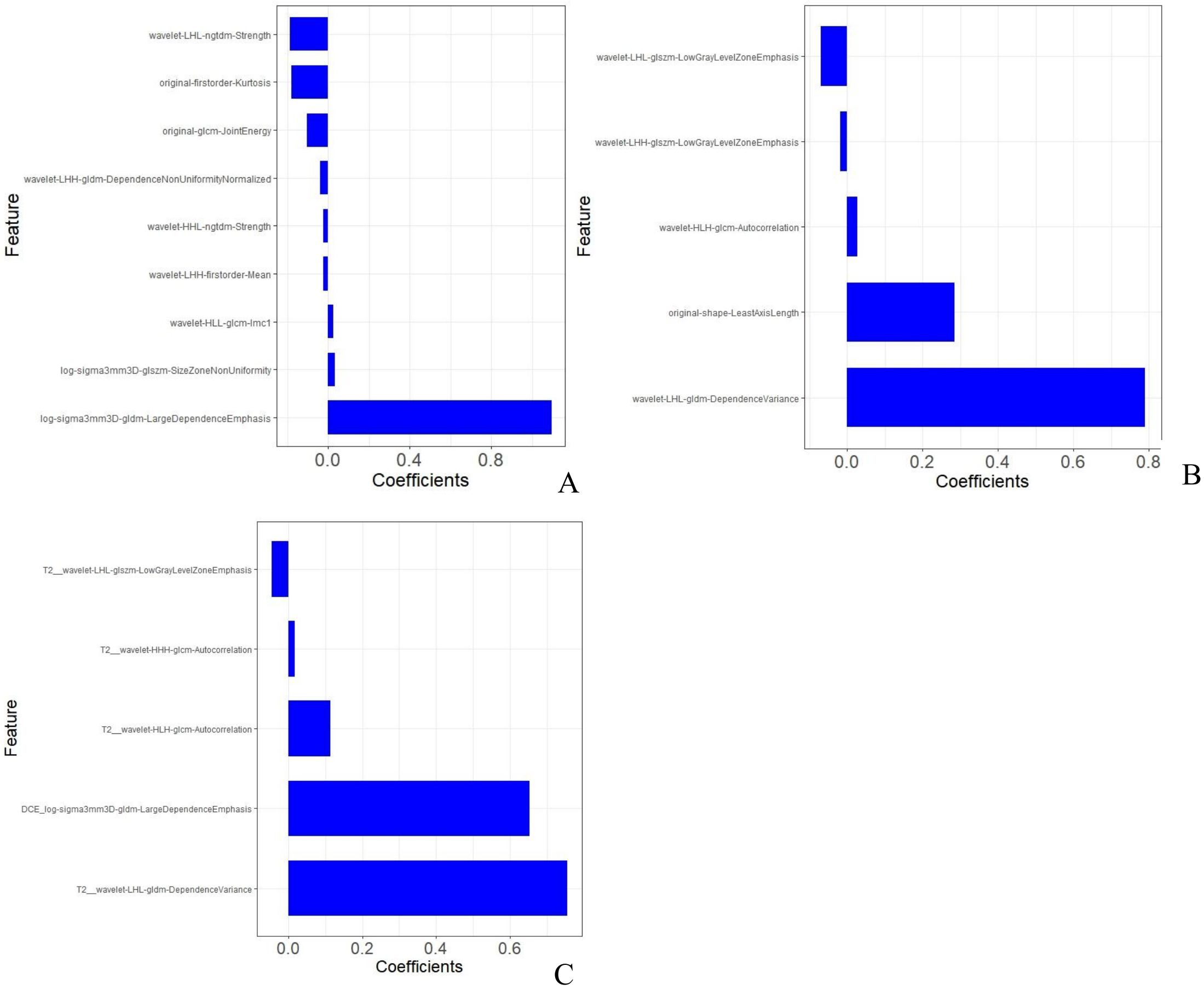

Supplementary Fig.1 The retained radiomics features and corresponding coefficients of the DCE-MRI-based model (A), the FS-T2WI-based model (B) and the fusion sequence model (C) after performing LASSO regression analysis.

Supplementary Table S1 Protocol parameters of different sequences

Sequence	TR (ms)	TE(ms)	FOV (mm)	matrix size	slice thickness (mm)	b vaule
T1WI	7.65	2.34	340×340	448×336	0.8	
FS-T2WI	6710	75	340×340	320×256	4	
DWI	3400	71	340×340	168×168	5	50，400，800
DCE-MRI	4.66	1.68	360×360	384×296	1.6	

Supplementary Table S2 Comparison of clinical and MRI features between training and validation cohorts

Characteristics	Training cohor（n=155）	validation cohort（n=66）	P
Age [$M(P_{25}, P_{75})$]	49.00 (42.00, 57.00)	50.00 (42.50, 58.50)	0.756
Lymphocyte [$\times 10^9$/L, $M(P_{25}, P_{75})$]	1.78 (1.39, 2.04)	1.73 (1.45, 1.97)	0.651
Neutrophil [$\times 10^9$/L, $M(P_{25}, P_{75})$]	3.22 (2.62, 3.99)	3.12 (2.61, 3.77)	0.331
Monocyte [$\times 10^9$/L, $M(P_{25}, P_{75})$]	0.42 (0.35, 0.50)	0.42 (0.33, 0.46)]	0.344
Platelet [$\times 10^9$/L, $M(P_{25}, P_{75})$]	203.00 (165.00, 248.00)	190.50 (158.25, 240.50)	0.311
LMR [$M(P_{25}, P_{75})$]	4.24 (3.42, 5.24)	4.25 (3.51, 5.22)	0.632
NLR [$M(P_{25}, P_{75})$]	1.82 (1.36, 2.44)	1.78 (1.37, 2.19)	0.654
PLR [$M(P_{25}, P_{75})$]	114.38 (90.91, 144.27)	113.14 (87.57, 143.82)	0.747
SII [$M(P_{25}, P_{75})$]	364.33 (270.70, 482.85)	343.51 (255.77, 450.46)	0.372
ER			1
Negative	49.00 (42.50, 57.00)	50.00 (41.25, 58.50)	
Positive	49.00 (42.50, 57.00)	50.00 (41.25, 58.50)	
PR			0.587
Negative	65 (41.9)	31 (47.0)	
Positive	90 (58.1)	35 (53.0)	
HER2			
Negative	106 (68.4)	49 (74.2)	0.478
Positive	49 (31.6)	17 (25.8)	
Histological grade			
I	42(27.1)	16(24.2)	0.907
II	72(46.5)	32(48.5)	
III	41(26.5)	18(27.3)	
Ki-67 percentage	30.00 (15.00, 50.00)	30.00 (15.00, 50.00)	0.858
Tumor size[mm, $M(P_{25}, P_{75})$]）	27.44 (18.34, 36.82)	25.24 (16.76, 33.50)	0.214
Tumor morphology [n(%)]			1
Regular	74 (47.7)	32 (48.5)	
Irregular	81 (52.3)	34 (51.5)	
Tumor location [n(%)]			0.34
Left	74 (47.7)	32 (48.5)	
Right	81 (52.3)	34 (51.5)	
Tumor quadrant [n(%)]			0.264
Outer upper	47 (30.3)	27 (40.9)	
Outer lower	41 (26.5)	21 (31.8)	
Upper inner	29 (18.7)	7 (10.6)	
Lower inner	19 (12.3)	5 (7.6)	
Others	19 (12.3)	6 (9.1)	
TIC [n(%)]			0.975
I	7 (4.5)	3 (4.5)	
II	47 (30.3)	21 (31.8)	
III	101 (65.2)	42 (63.6)	
BI-RADS [n(%)]			0.961

<4	7 (4.5)	2 (3.0)	
4a	26 (16.8)	12 (18.2)	
4b	38 (24.5)	16 (24.2)	
4c	42 (27.1)	20 (30.3)	
5	42 (27.1)	16 (24.2)	
Enhanced pattern [*n*(%)]			0.925
Mass-like	103 (66.5)	45 (68.2)	
Non-mass-like	52 (33.5)	21 (31.8)	
MRI-ALN [*n*(%)]			0.153
Negative	103 (66.5)	45 (68.2)	
Positive	52 (33.5)	21 (31.8)	

LMR Lymphocyte monocyte ratio, NLR Neutrophil lymphocyte ratio, PLR Platelet lymphocyte ratio, SII Systemic immune inflammation index, ER oestrogen receptor, PR progesterone receptor, HER2 human epidermal growth factor receptor 2, TIC Time-signal intensity curve, BI-RADS Breast imaging reporting and data system, MRI-ALN ALN status on MRI
